# New Copper Alloys Used to Make Products Intended for Contact with Drinking Water

**DOI:** 10.3390/ma14216301

**Published:** 2021-10-22

**Authors:** Michał Chruściński, Szymon Szkudelski, Jacek Borowski, Artur Meller, Marcin Suszyński

**Affiliations:** 1 The Łukasiewicz Research Network—Metal Forming Institute, 61-139 Poznań, Poland; michal.chruscinski@inop.poznan.pl (M.C.); szymon.szkudelski@inop.poznan.pl (S.S.); borowski@inop.poznan.pl (J.B.); 2 Faculty of Mechanical Engineering, Poznan University of Technology, 60-965 Poznań, Poland; artur.meller@doctorate.put.poznan.pl

**Keywords:** lead-free copper alloys, flashless forging, precise forging, lead

## Abstract

This article presents the results of tests conducted as part of a research project with the primary objective of developing new copper alloys with limited lead content. The new group of materials were created in a production plant. As part of tests, a group of 22 alloys were selected for testing in castability, structural characteristics and hardness. Based on the test results obtained, the group of alloys under study was narrowed down to nine. The mechanical properties of these alloys were determined in static tensile tests as well as in uniaxial upsetting tests at elevated temperature, on the basis of which the group of alloys under investigation was further narrowed to three. Further studies involved technological verification of the application of these alloys under industrial conditions. These alloys were subject to numerical forging analyses, along with forging tests, under semi-industrial conditions, where the degree of filling of a die impression at a specific temperature was measured using an optic scanner. The quality of production of the obtained forgings was evaluated macroscopically with simultaneous observations of the microstructure.

## 1. Introduction

The main goal of the work was to produce ecological brass that would exhibit better plastic properties and machinability, as well as a lower content of harmful chemical elements that negatively affect the quality of drinking water. Improved plastic properties are particularly important in hot forging processes, for example in the production of plumbing fixtures for drinking water. Nowadays, in order to be able to fully fill the commercial brass, such as ‘Ecobrass’, void, it is imperative to raise the forging temperature to over 800 °C, which, in conditions of traditional forging efficiency (30–50 pieces/min), triggers rapid processes of thermoplastic wear of the forging tools. As a result, the forges are compelled to reduce their efficiency in order not to damage the tools. This is one of the major factors influencing the market prices of the fixtures. For flashless forging, it is required to use forging tools with superior strength in comparison to their traditional forging analogs [1,2,3]. During flashless forging, there may be difficulties filling the forging impression, for example the narrow ribs or sharp corners, which may then lead to excessive loading and cracking of the material [4,5]. The results of the research on lead-free alloys have been presented in experiments [6,7,8] on the influence of selected elements on corrosion, microstructure and machinability.

The issue of production efficiency is also very important during the machining of forged fixtures. The better the machinability, the greater the adjustability of the machining parameters. When modifying the chemical composition, special attention should be paid to the toxicity of the alloy components. The objective of this paper was to determine the influence of selected alloying agents, as well as the impact that the morphology of the microstructure of developed alloys has on machinability and corrosion resistance, which in turn determines the washout of harmful elements into drinking water. The solution to the problem that is being applied at this point in time is the production of a variety of silicon-containing brasses with more complex chemical compositions [3]. An example of such a silicon brass developed and marketed in recent years is EcoBrass^®^ (Tokyo, Japan), which is manufactured both as a casting alloy in the form of pigs and as a product subjected to plastic deformation. It is characterized by very good castability (considerably better than the castability of CuSn5Zn5Pb5), as well as high strength, favorable machinability (thanks to the Cu8Zn2Si and Cu4ZnSi stages in the matrix of the α phase of the microstructure), very good resistance to dezincification and stress corrosion and, above all, by very good suitability for hot plastic deformation. Despite their many advantages and very encouraging descriptions and specifications provided by their manufacturers, currently available commercial lead-free alloys have many flaws: poorer castability leading to significantly worse casting tightness and their corresponding high price (according to various sources, between 25% and 50% higher than that of lead-containing alloys). In the case of alloys with added selenium, the evident toxicity of the latter may pose problems in the future. In the case of alloys containing silicon applied in the manufacture of products galvanically coated with chromium, significant problems associated with hard inclusions may arise, too.

The demand for copper alloys with low lead content, or completely devoid of it, in the manufacture of products intended for use as drinking water vessels arises from, among other things, legal and administrative issues in the United States and the European Union. The three fundamental documents pertaining to this are the “Reduction of the Lead in Drinking Water Act” No. 3874 of the US Congress, which was passed in 2011 and restricts the content of lead in drinking water; European Union Directive No. 98/83/EC on the “Quality of Water Intended for Human Consumption”, amended in 2015, and the Common Approach of 2011 adopted by Germany, France, the Netherlands and the United Kingdom (4 MSI) on the “Acceptance of Metals Used for Products that Come into Contact with Drinking Water”, amended in 2016. The last directive defines the array of metals to be used with drinking water. Such materials are copper alloys containing added bismuth and mischmetal as a modifier. They were developed and patented in the USA by Singh [9,10] and marketed under the brand name Federalloy^®^ (Bedford, OH, USA) by Federal Metal Company, Bedford, OH, USA. This group covers over 15 alloys (different types of bronzes and brasses) which are currently quite common in the US and other countries. The appropriate mechanical properties and machinability are achieved thanks to the simultaneous addition of bismuth and mischmetal as a modifier or an equivalent admixture of rare-earth metals. According to reports published by the manufacturer of these alloys [11,12], they contain less than 0.10% lead and have technical and technological characteristics similar to that of the lead-containing alloys that have been used until date. They are also distinguished by a better microstructural homogeneity than alloys containing Pb and they exhibit very good mechanical grinding, polishing and galvanic coating characteristics. Copper alloys in which bismuth and selenium are simultaneously applied as additives constitute a separate group of materials. These alloys are the result of an extensive research program implemented by a consortium headed by the American Foundry Society, in response to curbs on the amount of lead permissible in drinking water, which was introduced in the USA [13]. The alloys have found their commercial applications as a group of alloys initially named SeBiLoy^®^ (Salt Lake City, UT, USA) and currently sold under the EnviroBrass^®^ (Salt Lake City, UT, USA) brand name. The simultaneous application of bismuth and selenium additives comes from the fact that their combined beneficial impact on machinability is stronger than the aggregate total of their separate effects [14,15]. Bismuth inhibits washouts of selenium into water, which is particularly important considering the harmfulness of the latter and the need to limit its content in drinking water. While the beneficial effects of these alloying agents are being employed, other grades of casting alloys have also been developed [16,17]. However, selenium as an alloying agent (in the production of these amalgams) that generates toxic smoke, which is also why it has proven to be more favorable as a bismuth selenide bath [18,19]—a compound specially produced by Asarco Company as an additive in the manufacture of EnviroBrass^®^ alloys. The EnviroBrass^®^ group of alloys comprises three casting alloys, of which the first two are zinc- and tin-containing bronzes, besides bismuth and selenium, while the third is a brass additionally containing tin and aluminum components. As a result of the quest for alloys that could replace lead-containing bronzes and brasses, work on the application of alloys containing silicon is now at an advanced stage. Studies have shown that silicon brass CuZn14Si4 can be a likely alternative to CuSn5Zn5Pb5 [20], while silicon bronzes like CuZn5.5Si4.5 and CuZn14Si4 can serve as substitutes for bronzes like CuZn5Sn5Pb5 and CuZn9Sn3Pb7 [21,22]. Alloys containing silicon have comparable elevated castability values, as well as greater levels of hardness and strength. They, however, have slightly poorer, yet acceptable, machinability, comparable corrosion resistance, and poorer solderability. They are distinguished by their relatively good suitability for surface finishing treatment (grinding, polishing, slightly poorer adhesion to galvanically applied coatings). The main disadvantage of alternative CuZnSi alloys is their significant higher price, which is mainly due to the lack of sources of cheap scrap that can be used for their production. In view of that, this paper presents the results of research work aimed at finding new alloys that will serve as alternatives to those already present on the market. Most products intended to come into contact with drinking water are manufactured using hot forging technology by means of flash or flashless forging.

## 2. Materials and Methods

### 2.1. Materials

The studies focused on issues relating to the selection of particular chemical compounds, as well as to the melting and casting parameters of a new group of alloys and the evaluation of their technical, technological and functional properties. The scope of work performed on the selected group of alloys were melting tests, which were conducted in an induction crucible furnace with a capacity of 10 kg Cu and covered by dried charcoal. Alloys were placed in cast iron molds that had been pre-heated to approx. 300 °C. The rod ingots were 30 and 40 mm in diameter and approx. 300 mm in length. The stock materials used were the following metals: copper, zinc, tin, technically pure lead and preliminary alloys. The usage of the preliminary alloys is of paramount importance in cases where the boiling temperature of one ingredient is lower than the melting temperature of another, where the alloying agents differ substantially in specific gravity, where the mutual solubility is low, and where the ingredient with higher melting temperature has a much lower specific heat capacity than those with a lower melting temperature. Due to big differences in the melting temperatures of individual alloying agents and the limited mutual solubility of some of them, it was necessary to use preliminary alloys such as: CuAl50, CuFe20, CuNi15, CuSi16, CuAs7, CuB2, CuP17.7. The castability measurements of all developed alloys were taken by the spiral test casting method (W.Ruff) using an oiled fine-fraction molding mass. The molten metal’s ability to fill recesses in the mold and precisely reproduce its shape were evaluated based on the lengths of the spiral castings. Hardness measurements of the cast’s experimental alloys were performed using the Vickers method (HV10) with a Wolpert 2Rc universal hardness tester. The characteristics of the microstructure and morphology of the phases present in the alloys were observed under an Olympus GX71F light microscope while making use of STREAM image acquisition and analysis software. The mechanical properties of selected experimental alloys were determined by tensile testing using an Instron 4505/5500K strength tester. Evaluations of mechanical properties at elevated temperatures were carried out based on uniaxial compression testing conducted within a 650–800 °C temperature range. The upset forging test was conducted using an Instron 4505/5500K strength tester by heating specimens in an open furnace and soaking them for 15 min (after the set temperature was reached). Specimens with a H0/D0 slenderness ratio equal to 1.5 were used in the upset forging. The upset forging was performed using anvils with working surfaces made of sintered tungsten carbide inserts (G50S carbide) measuring up to approx. 3.5 mm specimen height (H). This corresponded to a deformation of approx. 75% (draft ε = ln(H/H0) approx. 1.5). Analyses of upset forging curves were conducted taking into account the sag of the machine as well as elements of the force transmission chain.

### 2.2. Forging

In order to assess the chances of achieving the intended copper alloy grades, simulations of their bulk formation were conducted. The simulations were carried out in Q-Form software, which does numerical analysis of the bulk (volumetric) forming process. Detailed process parameters were input into the software, i.e., the tool’s geometry, the press parameters and the parameters of the stock materials. The results of the material tests performed for the new copper alloys served as a supplement for the database of the deformed materials in the Q-Form software. Numerical analysis was conducted on the basis of “Cap 1” forging. A stock material temperature of 750 °C was agreed upon for the simulations. In order to verify the data obtained, forging tests on the developed materials were also carried out using a test stand consisting of a press with a 2000 kN nominal force and a 175-mm slider pitch. The press was rigged with the following: an automatic stock feeding system with a stock temperature control system, an automatic forging collection system, an automatic forging tool lubrication system, an automatic hydraulic pad pitch regulation system enabling precise regulations of the die set’s immersion vis-à-vis the forging punch. Tests on the influence of forging temperatures were carried out for “Cap 1” forging at temperatures of 650, 700 and 750 °C. The stock material was heated in a Nobertherm laboratory furnace with a setting accuracy of +/−1 °C. The stock was soaked for 30 min (after reaching the set temperature). An ATOS COMPACT SCAN 5M optical scanner with a measuring field of 110 × 150 mm and measurement uncertainty of 0.02 mm was used to evaluate shape accuracy. Analysis of the scans obtained was conducted with reliable GOM Inspect Professional 8 software. Metallographic observations were also carried out under a Nikon Eclipse L150 light microscope.

## 3. Results

### 3.1. Material

With regards to the acceptable levels of metals in drinking water, Directive DWD 98/83/EC [5] imposes significant restrictions (Table 1) on chemical parameters, as well as strict guidelines on organoleptic and physicochemical requirements.

The conditions discussed in the introduction of this article were reflected in the formulation of the following assumptions regarding materials that were decisive in the selection of the chemical compositions of the experimental alloys, namely:

- Compliance with the guidelines of 4 MS Common Composition List for materials from group B;
- Alloys from the group of biphasic α+β’ brasses;
- Good technological properties—suitability for forging and machinability;
- Good functional properties—resistance to corrosion;
- As low material cost as possible.

It is assumed that lowering the Pb content will primarily cause a significant deterioration in the machinability of the tested materials. The intended course of action is then to modify the chemical composition of experimental alloys. If the Pb level is lowered, the machinability should be improved by the use of additives influencing the morphology of the alloy microstructure with simultaneous research on the effect of heat treatment. The introduction of modifications in the scope of the chemical composition, as proposed below, was aimed at the fragmentation of the microstructure of the alloys by introducing aluminum and silicon additives that alter the limits of solubility of solid zinc in copper. Additionally, they cause a certain degree of strengthening of the alloy and change its corrosion characteristics. Summing up, when choosing a strategy to select the composition of alloys to forge elements that are intended make contact with drinking water, and which meet the current requirements of the legislation in the area of lead control, inclusion of the following groups of materials in the research is recommended:

- A low lead CuZnPb alloy with a Pb content below the current lower Pb level defined in 4MSCCL (below 1.6% Pb);
- A low lead CuZnPb alloy, DZR (dezincification-resistant), below the current Pb accepted level defined in the 4MSCCL (below 1.6% Pb), and which contains extra additives that purposefully modify the morphology of the microstructure and change its technical and technological characteristics.

For this reason, 22 alloys divided into three groups were selected for testing, the chemical composition of which is presented in Table 2, Table 3 and Table 4:

Due to the diversity of the properties of the individual elements, a specific method and sequence of their melting were developed. First, the components with maximum specific heat capacity, maximum melting and boiling temperature and solubility and minimal chemical interaction with the environment were melted. Analysis of the properties of individual elements demonstrated the need to prepare special preliminary alloys. The preliminary alloys were prepared in the form of CuAl50, CuFe20, CuNi15, CuSi16 and CuAs7, and preliminary alloys of the following grades were also used: CuB2, CuP14.7.

#### 3.1.1. The Castability Test

**Figure 1:**
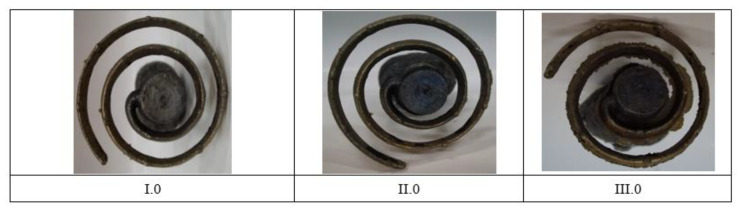
The looks of castability test spirals of representative metals from groups I–III. SJ 560–850 mm.

One of the most important technological parameters of alloys is their ability to fill the casting mold in the castability test. The mold filling temperature was 1010 °C. Examples of the test results for representatives of individual groups are presented in Figure 1. The castability of the developed alloys falls within the 510–860 mm range.

#### 3.1.2. Observations of the Ingot Macrostructure

**Figure 2:**
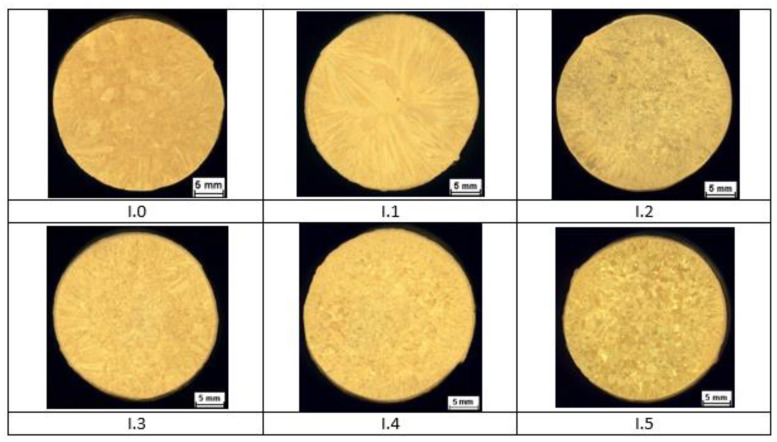
Ingot macrostructure of experimental alloys from group I.

**Figure 3:**
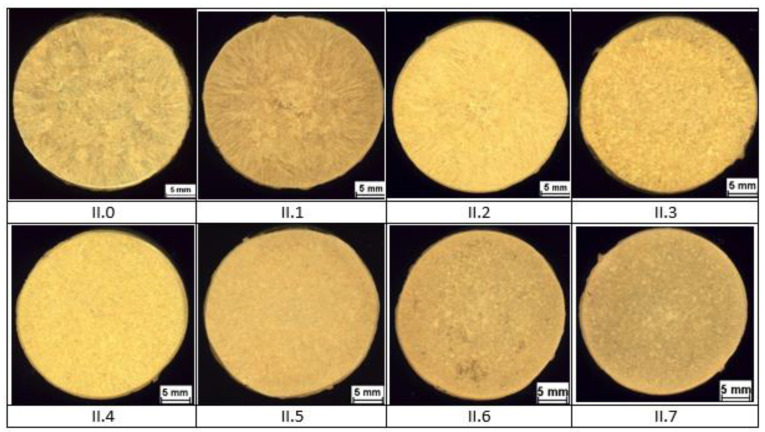
Ingot macrostructure of experimental alloys from group II.

**Figure 4:**
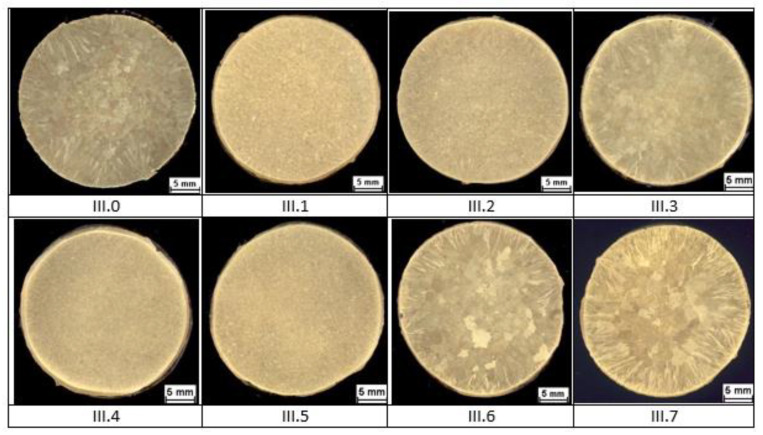
Ingot macrostructure of experimental alloys from group III.

The results of macrostructural observations of ø 30 × 330 mm ingots are given in Figure 2 for group I alloys, in Figure 3 for group II alloys, and in Figure 4 for group III alloys. No casting defects that could have precluded further testing of the material were discovered. Certain differences in the macrostructure of the cast bars were as a result of differences in the chemical compositions of the tested brass as well as differences in the solidification conditions of the ingots. It should be noted that a satisfactory fragmentation effect of the casting structure was not obtained due to the addition of boron.

#### 3.1.3. HV Hardness Measurement of Cast Ingots

HV10 hardness measurement of the experimental alloys was performed on ingot specimens in the form of ø 30 mm shafts following their grinding on sandpapers with the view to remove the potential effects of material deformation resulting from cutting. The measurement results are provided in Table 5.

#### 3.1.4. Selection of Alloys

The above test results served as the basis for selection of the alloys. Reference materials (alloys I.0, II.0 and III.0) and two more alloys from each group were selected for further expanded tests. In the case of group I alloys, due to the absence of a clear modification effect, alloys 1, 2 and 3 are very similar in terms of their chemical compositions (similar reduced lead content, a similar Zn/Cu content ratio). For this reason, only one of them was selected for further testing (alloys I.3). The second alloy was I.4. It contains an addition of aluminum and is characterized by a significant increase in hardness, but not up to that of alloy I.5. It was accepted that this alloy would be a good indicator of the influence of aluminum on the characteristics of this group of brasses. In the case of group II, brasses II.1 and II.2 (with reduced Pb content and a similar Zn/Cu content ratio) did not exhibit any clear effects of the influence of B microadditives either. It was therefore decided to continue the tests with only one of them (II.2). The selection of the next material (alloy II.5) was dictated by the objective to assess the influence of the addition of silicon on the investigated characteristics (Si had the strongest influence on shifting the boundaries of the solubility of zinc in copper.). The influence of the AI additive will be studied taking alloy I.4 as an example. In the case of group III (with enhanced resistance to dezincification), addition of equally reduced Pb and the same addition was used in all alloys. The addition of zinc (Zn/Cu content ratio) was differentiated in the experimental alloys in order to obtain a diverse microstructure in terms of the amount of the β’ phase in the microstructure, and similarly to the previous group of brasses, an attempt was made to refine the casting microstructure through micro-additive B. The result of modification did not meet our expectations and so, alloys III.1 and III.6 were chosen for further testing. They contain similar alloying agents but vary in their Zn/Cu ratio.

#### 3.1.5. The Mechanical Properties of the Cast Ingots

The highest tensile strength Rm was noted for alloys I.4 and II.5—428 MPa and 464 MPa, respectively, and elongation A levels—26% and 33.8%, respectively. The lowest tensile strength was noted for alloys III.0 and III.6—at 325 MPa and 308 MPa, respectively, while their elongations A were 51.4% and 43.5%, respectively. The other alloys had tensile strengths Rm ranging between 355 and 370 MPa and elongations A in the 36–43% range. 

#### 3.1.6. The Upset Forging Curves of the Experimental Alloys

**Figure 5:**
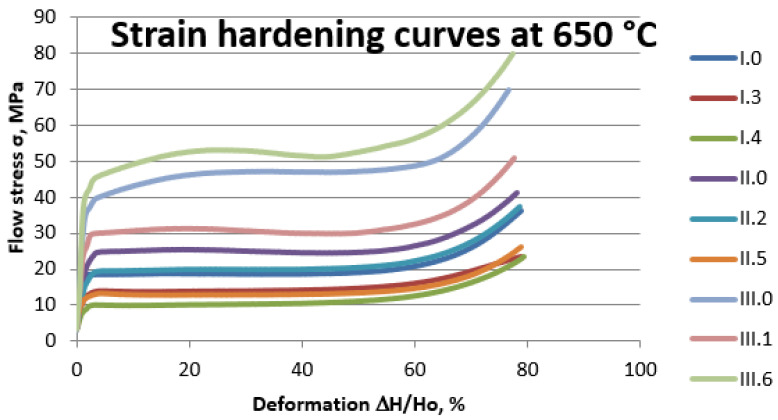
Strain hardening curves of the experimental alloys at temperature 650 °C.

**Figure 6:**
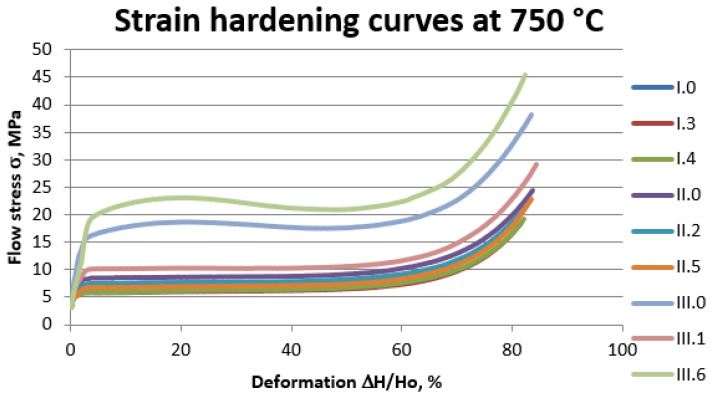
Strain hardening curves of the experimental alloys at temperature 750 °C.

Selected results of forging tests for the experimental alloys at individual upset forging temperatures are shown for temperature 650 °C (Figure 5) and for temperature 750 °C (Figure 6).

Ultimately, three alloys with their chemical compositions given in Table 6 were selected for further tests including forging. For these three alloys, molten metals that made it possible for forging tests to be carried out under semi-industrial conditions, were prepared. 

### 3.2. Forging

#### 3.2.1. Numerical Analysis

**Figure 7:**
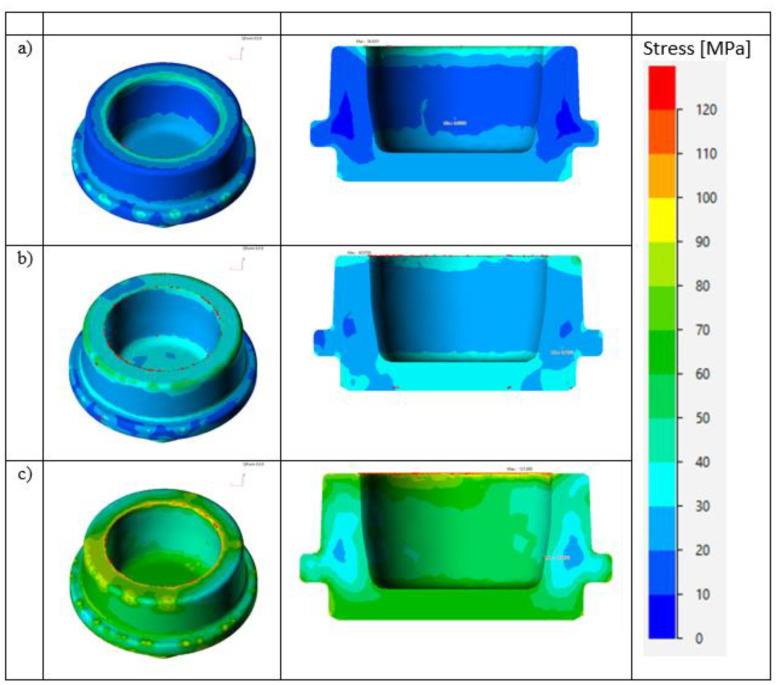
Distribution of real stresses in “Cap 1” forging at a formation temperature of 750 °C: (**a**) alloy 1, (**b**) alloy 2, (**c**) alloy 3.

Simulations of “Cap 1” forging were carried out for the three selected copper alloys. A comparison of the stress distribution in the three material variants is shown in Figure 7. The mesh properties of the simulations have been given below. 

Surface elements: 28,118; volumetric elements: 132,125; tools temperature: 250 °C; piece temperature: 750 °C; mechanical press: 2000 kN; press stroke: 175 mm; number of press strokes: 110 per minute.

**Figure 8:**
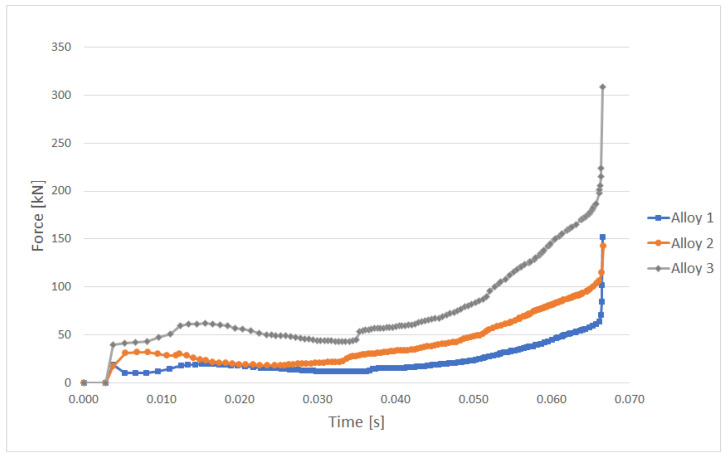
Charts of formation forces acting on “Cap 1” forging at temperature 750 °C—3 alloys.

Charts of formation forces acting on “Cap 1” forging that were registered during numerical simulation at temperature 750 °C for selected copper alloys are presented in Figure 8.

#### 3.2.2. Forging Tests

**Figure 9:**
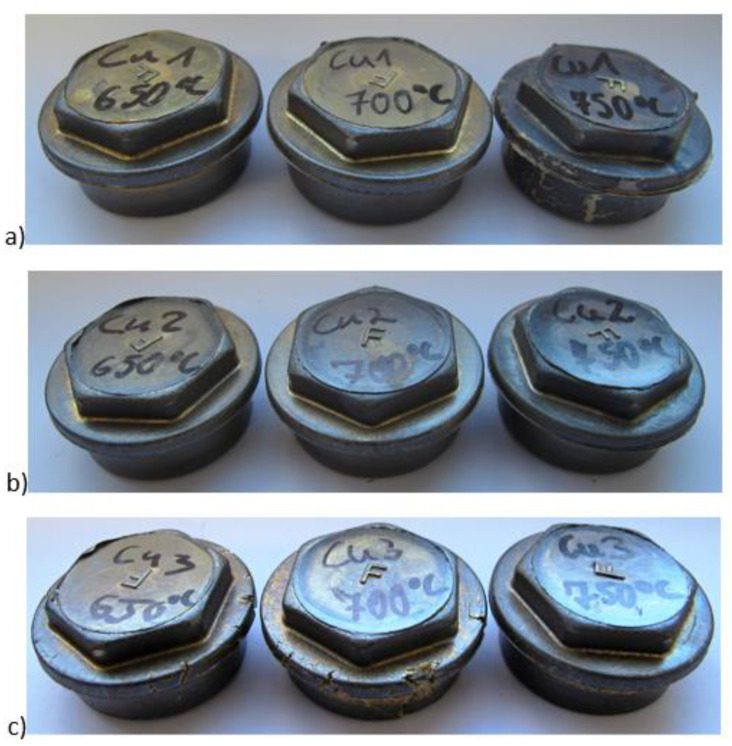
“Cap 1” forgings made of new alloys formed at different temperatures. Alloys: (**a**) alloy 1, (**b**) alloy 2, (**c**) alloy 3.

In order to assess chances of forming selected copper alloys, “Cap 1” forging tests were carried out at temperatures 650, 700 and 750 °C. The forgings obtained are shown in Figure 9. Material tests were performed on the forgings obtained.

**Figure 10:**
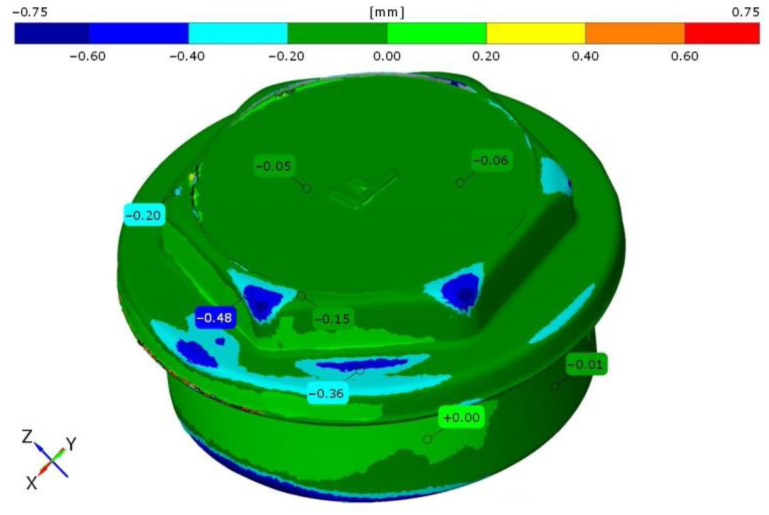
The influence of stock material on the forging shape, insufficient volume of the stock.

After all forgings were forged, a scan was performed using an optical scanner in order to ascertain if the die impression had been correctly filled. An example color map of a forging process obtained at 750 °C from alloy 3 is presented in Figure 10. The 3D model of the forging was accepted as the reference for comparison with the produced forgings.

#### 3.2.3. Metallographic Analysis

**Figure 11:**
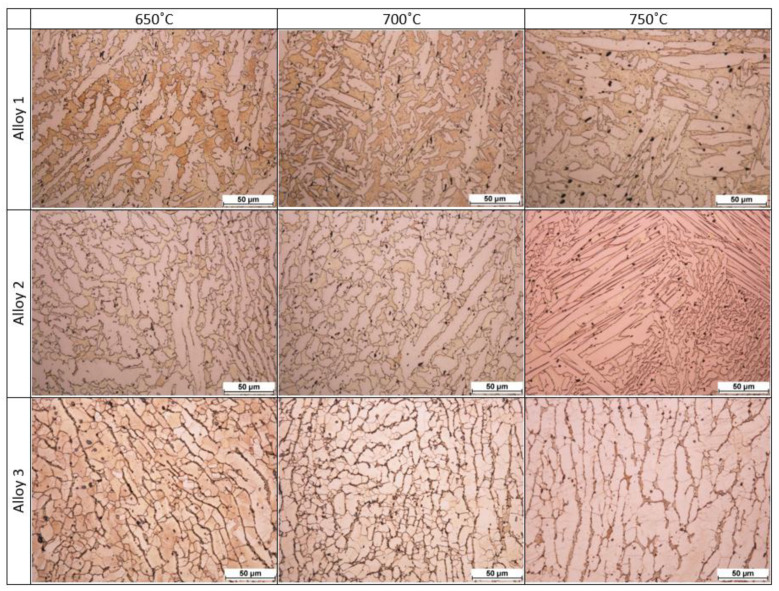
Microstructure of forgings made using newly developed alloys with reduced lead content.

As part of material tests, microstructural observations were carried out for the three copper alloys. Metallographic tests of the specimens were carried out in order to detect and determine their characteristic structural features. Figure 11 presents photographs of the microstructures.

## 4. Discussion

Analysis of documents related to imposed legal restrictions concerning the washing- off of lead into drinking water and the production requirements associated with this problem made it possible to determine the chemical compositions of the 22 experimental alloys that were the objects of study. Conducted casting tests (Figure 1) made it possible to obtain a material consistent with our expectations regarding its chemical composition. The spiral casting tests demonstrated good castability in all tested alloys. Certain experimental alloys containing 1% Si (II.6 and II.7) were distinguished by very good fluidity, reaching a spiral length of over 800 mm. The results obtained served as the basis for the selection of alloys and the continuation of their testing with respect to the nine selected materials representing three groups of brasses. The microstructure of the tested alloys (Figure 2, Figure 3 and Figure 4) belongs to the same type of brasses in all cases, i.e., α+β’ brasses, with lead particles also present. Hence, it is consistent with the type assumed at the outset, making it possible to obtain good suitability for hot plastic deformation. Hardness measurements (Table 2) of ingots indicate that the applied maximum additions of aluminum and silicon significantly increase hardness. In extreme cases (alloys II.6 and II.7), the hardness of the alloys amounted to more than 160 HV10 (for casting), i.e., as much as brasses in their hardened state attain. The ingot cooling rates, which were not the same, certainly also had an effect on hardness value. In alloy group I, the experimental brasses with modified chemical composition are characterized by slightly lower values of plastic flow stress (Figure 5 and Figure 6) for all of the tested temperatures. A comparison of materials in this group hints that experimental alloys should also exhibit very good suitability not only in the scope of extrusion but also in hot forging. Similar results were obtained for group II alloys, in which experimental brasses were also distinguished by slightly better parameters with regard to plastic flow stress level for the tested temperature range. Furthermore, the rate of cooling following plastic deformation may also be a significant parameter for this group of alloys, as indicated by observations of the specimens’ microstructure following upset forging. Group III alloys are characterized by a markedly higher level of plastic flow stress. Measurements of the mechanical properties of the alloys showed slightly higher strength properties in those with modified compositions as compared to reference alloys, and slightly lower plasticity (elongation), with the exception of alloy III.6. Obtained data on the mechanical properties are consistent with the requirements of standard PN-EN 12165 (except for one: reference alloy II.0 had a slightly higher Rm value). However, one needs to keep in mind that normative requirements pertain to materials intended for forging, and that the experimental alloy tests were performed on materials following static casting and so may be slightly undervalued. Preliminary evaluation of suitability for plastic formation, based on the upset forging test, showed relatively good suitability for hot plastic formation. The least favorable properties in this scope were displayed by group III alloys, which was due to their slightly different phase composition. In group I alloys, the experimental brasses with modified chemical composition were characterized by slightly lower values of plastic flow stress for all of the tested temperatures. A comparison of materials in this group showed that experimental alloys should also exhibit very good suitability not only in extrusion, but also in hot forging.

It can be seen from the obtained results of numerical simulations that, for group III alloys, the real stress values attained were two times higher than those obtained in the other two materials. The higher stresses translate into a greater force required during the forging process, which is about double the case of other alloys.

During real-life assessments on test stands, “Cap 1” forging using the three new alloys were also verified. The forging temperature was set at 650, 700 and 750 °C, as was the case in the numerical simulations. Alloys 1 and 2 achieved full filling of the die impression at each of the studied temperatures. Alloy 3 did not achieve filling of the impression in any of the studied temperatures. Cracks could be observed on the surface of the forgings made from alloy 3, which were associated with the substantially higher temperature range of plastic formation of this alloy compared to the other two. Cracks were observed in alloy 3 at each of the studied temperatures. Analysis of the microstructure revealed that alloys 1, 2 and 3 were characterized by a biphasic microstructure: crystals of the alpha solid solution (copper) and dark crystals of the beta solid solution (zinc) were visible. Elongated grains similar to dendritic grains were present in alloy 1. In alloy 1, slight grain growth occurred at 750°C. Elongated grains were mostly present in alloy 2 at 750 °C. Changes in grain elongation in different directions occurred at 650 °C and 700 °C. Grains of the alpha phase were more elongated than in alloy 1. At 750 °C, there was a visible absence of grain elongation indicating recrystallization of the material. In alloy 3, crystals of the solid solution were partially twinned, with an elongated beta’ phase on the boundary of the alpha phase grains. At 650 °C, a fine-grained microstructure was visible, whereas at 700 °C and 750 °C, the grains were elongated with local clusters of fine grains.

## 5. Conclusions

The conducted research made it possible for the chemical compositions of alloys with low lead content for three groups of materials to be determined. After a detailed analysis of the obtained results, it was possible to select the three most promising new copper alloys for further research and verify them during forging trials. The tests carried out on a batch of experimental alloys can be summarized as follows:

- The castability of alloys is fully satisfactory from the standpoint of casting ingots for extrusion. With reference to some alloys, it can be stated that they are characterized by very good casting properties, enabling them to be used as casting alloys.
- Mechanical properties in the cast state fulfill the requirements of stock materials intended for forging processes. This shows full feasibility of obtaining the required mechanical properties of the rods after hot plastic deformation.
- The developed alloys indicate that they are sufficiently suitable for hot plastic deformation, enabling the attainment of a good quality drawpiece. At high rates of deformation on the press, it was demonstrated that alloys 1 and 2 were characterized by good deformability. For alloy 3, the forging temperature during tests was too low, causing cracks and incomplete forgings.

The available data indicate that the problem of lead in drinking water, despite the passage of time, is still real and unresolved. Considerable lead toxicity, even if associated with low human exposure to its low concentrations in drinking water, can cause a broad array of health disorders in humans, especially in children [23,24,25]. For this reason, the authors propose the adoption of an appropriate legal framework that would regulate the amount of lead in brass alloys as follows:

The first stage of changes (restrictions) would be the introduction of “ecological varieties” of brass already in use (full compliance with PN-EN 12165 as well as with 4MSCCL) while capping the maximum limit of Pb at 2.2% and that of Ni at 0.1%.

The second stage of changes (constraints) would be to introduce further restrictions: Pb max. 1.6% with the inclusion of Al (and possibly others), and possible modification of the microstructure.

The third stage of changes (limitations) would be completely lead-free, two-component alloys with a modified composition and a microstructure with clearly aggravated machinability properties.

## Figures and Tables

**Table 1:** Selected requirements regarding metal levels in water intended for human consumption.

Chemical parameters—acceptable levels of certain metals									
Sb	As	B	Cr	Cd	Cu	Ni	Pb	Hg	Se
5.0 μg/L	10 μg/L	1.0 mg/L	50 μg/L	5.0 μg/L	2.0 mg/L	20 μg/L	10 μg/L	1.0 μg/L	10 μg/L
Organoleptic and physicochemical requirements as well as additional requirementsacceptable levels of certain metals									
Al.		Mn		Na	Fe	Mg		Ag	
200 μg/L		50 μg/L		200 mg/L	200 μg/L	7–125 mg/L		0.010 mg/L	

**Table 2:** Assumed chemical composition of alloys from group I.

Chemical Element/Designation of the Sample	Content of a Chemical Element, %					
	I.0	I.1	I.2	I.3	I.4	I.5
Zn	rest					
Al	0.04	0.04	0.04	0.04	0.50	0.90
Fe	0.25	0.25	0.25	0.25	0.25	0.25
Pb	2.0	1.5	1.4	1.4	1.4	1.4
Sn	0.20	0.20	0.20	0.20	0.20	0.20
Ni	0.25	0.10	0.10	0.10	0.10	0.10
B	-	-	0.001	0.002	-	-
P	0.05	0.05	0.05	0.05	0.05	0.05
Cu	59.0	59.0	59.0	59.0	59.0	59.0

**Table 3:** Assumed chemical composition of alloys from group II.

Chemical Element/Designation of the Sample	Content of a Chemical Element, %							
	II.0	II.1	II.2	II.3	II.4	II.5	II.6	II.7
Zn	rest							
Si	-	-	-	-	-	0.50	1.00	1.00
Al	0.04	0.04	0.04	0.90	0.90	0.04	0.04	0.04
Fe	0.25	0.25	0.25	0.25	0.25	0.25	0.25	0.25
Pb	2	1.5	1.5	1.3	1.3	1.1	0.7	1.1
Sn	0.2	0.20	0.20	0.20	0.20	0.20	0.20	0.20
Ni	0.25	0.10	0.10	0.10	0.10	0.10	0.10	0.10
B	-	-	0.002	-	0.002	-	-	0.002
P	0.05	0.05	0.05	0.05	0.05	0.05	0.05	0.05
Cu	60	59.5	59.5	60.0	60.0	59.5	60.0	60.0

**Table 4:** Assumed chemical composition of alloys from group III.

Chemical Element/Designation of the Sample	Content of a Chemical Element, %							
	III.0	III.1	III.2	III.3	III.4	III.5	III.6	III.7
Zn	rest							
As	0.10	0.10	0.10	0.10	0.10	0.10	0.10	0.10
Al	0.04	0.80	0.80	0.80	0.80	0.80	0.80	0.80
Fe	0.25	0.25	0.25	0.25	0.25	0.25	0.25	0.25
Pb	2.00	1.40	1.40	1.40	1.40	1.40	1.40	1.40
Sn	0.10	0.10	0.10	0.10	0.10	0.10	0.10	0.10
Ni	0.15	0.15	0.15	0.15	0.15	0.15	0.15	0.15
B	-	-	-	-	0.001	0.002	0.001	0.002
P	0.05	0.05	0.05	0.05	0.05	0.05	0.05	0.05
Cu	62.0	63.0	65.0	64.0	64.0	65.0	65.0	64.0

**Table 5:** Hardness measurements of the alloys under study.

ALLOYS FROM GROUP I	Alloy no.							
	I.0	I.1	I.2	I.3	I.4	I.5		
Average HV10 Hardness	94.2	105	103	106	119	149		
Std. Dev.	1.10	1.38	2.19	3.22	2.83	6.04		
ALLOYS FROM GROUP II	Alloy no.							
	II.0	II.1	II.2	II.3	II.4	II.5	II.6	II.7
Average HV10 Hardness	91.2	96.4	95.3	117	123	127	164	161
Std. Dev.	1.52	1.44	1.35	3.96	1.89	2.37	2.29	2.99
ALLOYS FROM GROUP III	Alloy no.							
	III.0	III.1	III.2	III.3	III.4	III.5	III.6	III.7
Average HV10	77.9	95.8	90.9	83.1	93.0	95.6	80.5	82.3
Std. Dev.	2.77	1.96	1.33	0.38	1.23	3.21	2.70	0.76

**Table 6:** The chemical compositions of copper alloys selected for further tests.

Alloy	Cu	Zn	Pb	Al	As	Fe	Ni	Sn
1	58.5	remainder	0.95	0.3	-	max 0.2	max 0.1	max 0.2
2	59.5	remainder	0.95	0.3	-	max 0.2	max 0.1	max 0.2
3	63.5	remainder	0.95	0.6	0.10	max 0.2	max 0.1	max 0.2

## Data Availability

Not applicable.
